# NGF and proNGF Regulate Functionally Distinct mRNAs in PC12 Cells: An Early Gene Expression Profiling

**DOI:** 10.1371/journal.pone.0020839

**Published:** 2011-06-03

**Authors:** Mara D'Onofrio, Francesca Paoletti, Ivan Arisi, Rossella Brandi, Francesca Malerba, Luisa Fasulo, Antonino Cattaneo

**Affiliations:** 1 Neurotrophic Factors and Neurodegenerative Diseases Unit, EBRI-European Brain Research Institute, Rome, Italy; 2 Neurogenomics IIT Unit, EBRI-European Brain Research Institute, Rome, Italy; 3 Scuola Normale Superiore, Pisa, Italy; University of Florida, United States of America

## Abstract

The biological activities of NGF and of its precursor proNGF are quite distinct, due to different receptor binding profiles, but little is known about how proNGF regulates gene expression. Whether proNGF is a purely pro-apoptotic molecule and/or simply a “less potent NGF” is still a matter of debate. We performed experiments to address this question, by verifying whether a proNGF specific transcriptional signature, distinct from that of NGF, could be identified. To this aim, we studied gene expression regulation by proNGF and NGF in PC12 cells incubated for 1 and 4 hours with recombinant NGF and proNGF, in its wild-type or in a furin-cleavage resistant form. mRNA expression profiles were analyzed by whole genome microarrays at early time points, in order to identify specific profiles of NGF and proNGF. Clear differences between the mRNA profiles modulated by the three neurotrophin forms were identified. NGF and proNGF modulate remarkably distinct mRNA expression patterns, with the gene expression profile regulated by NGF being significantly more complex than that by proNGF, both in terms of the total number of differentially expressed mRNAs and of the gene families involved. Moreover, while the total number of genes modulated by NGF increases dramatically with time, that by proNGFs is unchanged or reduced. We identified a subset of regulated genes that could be ascribed to a “pure proNGF” signalling, distinct from the “pure NGF” one. We also conclude that the composition of mixed NGF and proNGF samples, when the two proteins coexist, influences the profile of gene expression. Based on this comparison of the gene expression profiles regulated by NGF and its proNGF precursor, we conclude that the two proteins activate largely distinct transcriptional programs and that the ratio of NGF to proNGF *in vivo* can profoundly influence the pattern of regulated mRNAs.

## Introduction

NGF (Nerve Growth Factor), the prototype member of the neurotrophin protein family [1], is involved in the maintenance and growth of specific neuronal populations, both in the central and peripheral nervous system, through the interaction with two receptors: TrkA, a member of the Tyrosine Kinase receptors superfamily [2], and p75^NTR^, belonging to the Tumor Necrosis Factor (TNF) receptor superfamily [3].

**Figure 1:**
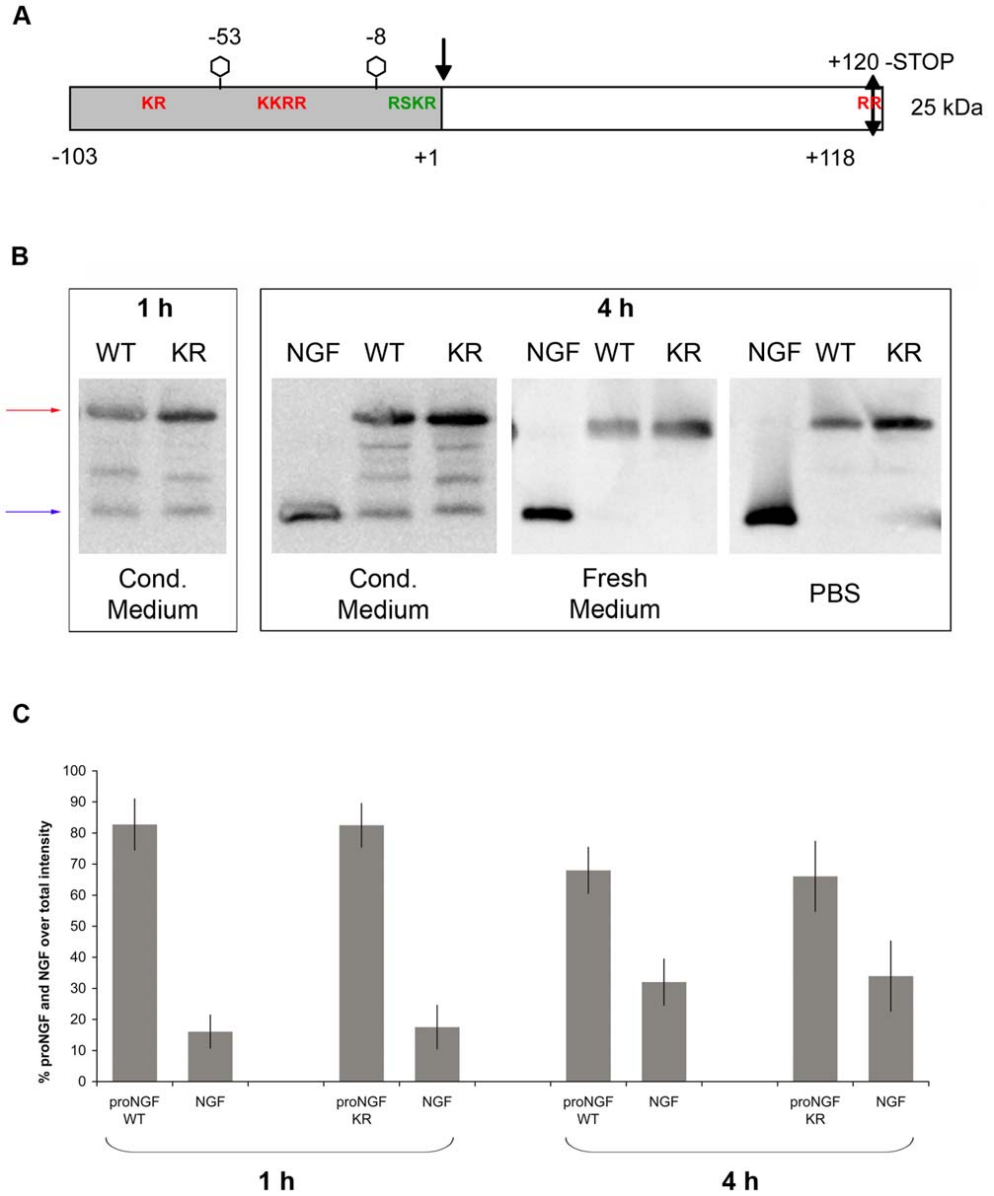
Description of proNGF and its stability. *Panel A: Schematic representation of the short form of proNGF*. The arrows mark the cleavage sites for furin, the double headed arrows represent the C-terminal processing site (post translational modification) and hexagons the potential N-glycosylation sites. In red, the di-basic amino acids that are important in the processing of the protein. In green, the consensus site for the cleavage by furin. In the present study, we obtained furin resistant mutants in this site (mutant proNGF-KR: RS**KR** to RS**AA** [52]). *Panel B: Western blot to test the stability of the proNGF samples in PC12 cells*. PC12 cells were plated and treated for 1 h or 4 h with 20 ng/mL of proNGF-WT or proNGF- KR or 10 ng/mL of NGF. Then, the medium was taken and 1 µg of recombinant proNGF or proNGF-KR or NGF was spiked into 50 µL of conditioned medium. Spiking controls in fresh medium and PBS were also carried out. The spiked medium was incubated at 37°C for 1 h or 4 h. The red arrow marks the band corresponding to the full length proNGF, the blue arrow marks the band corresponding to mature NGF. In the figure, WT stands for proNGF-WT and KR stands for proNGF-KR. *Panel C: Densitometric analysis performed on the Western Blot of the spiking experiments representing the percentage of proNGF proteolysis*. The bands corresponding to proNGF and NGF in the Western blot challenged with the anti-NGF antibody were quantified. The resulting intensities, normalized against the areas of the bands, were reported in the histogram. For each lane, corresponding to the different proNGF-WT or -KR treatments, the band intensities of proNGF and NGF, derived from proNGF proteolysis, were measured and the sum of the two bands intensities was assigned to a value of 100%. Among this total intensity, the intensity of the bands corresponding to proNGF and mature NGF was evaluated and expressed as %. The histogram is the result of the average of four independent experiments.

As all neurotrophins, NGF is translated as a pre-pro-protein [4]. In the case of NGF, two alternative translation initiation sites have been identified, leading to the formation of two precursor proteins, a long and a short form, respectively (Figure 1A), that are glycosylated *in vivo* [5]. The signal peptide is cleaved upon translocation into the endoplasmic reticulum, and the protein is further processed by furin protease in the trans-Golgi network [6], [7] or by extracellular proteases [8], [9], to give rise to mature NGF.

Besides its suggested roles as a precursor to mature NGF in the regulation of neurotrophin secretion [10] and as an intramolecular chaperone [11], [12], proNGF was found to display independent biological activities, different from those of its mature NGF counterpart, mediated by distinct, and somewhat complementary, receptor binding properties [13], [14]. In transfected cells and cultured neurons, proNGF was shown to induce p75^NTR^-dependent apoptosis [8], [15], [16], but also TrkA dependent neuronal survival, although less effectively than mature NGF [17]. proNGF is the predominant form of NGF in normal brain and its levels increase in the brain of patients affected by Alzheimer's Disease (AD) [18].

Sortilin, a member of the family of Vps10p-domain receptors, was identified as a specific receptor for proNGF [13] and proposed to control the sorting pathways of pro-neurotrophins [19]–[21]. The activation of the death signal by proNGF requires its interaction with both sortilin and p75^NTR^ receptors [13], [22]–[24]. The protein levels of proNGF, TrkA, p75^NTR^ and sortilin appear to be differently modulated in AD brains [25], [26].

The overall picture of neurotrophins activity, as precursors or as mature proteins, is therefore more complicated than previously thought. Whether proNGF is a true apoptotic molecule or a sort of “less potent NGF” is still a matter of debate [8], [17] and different biological outcomes of NGF and proNGF signalling have been suggested to depend on the ratio between NGF and proNGF [27], [28], on the presence of different kinds of proteases [9], [21], [29] and on the expression levels of the receptors [30].

It is therefore crucial to address the question of whether, and to what extent, NGF and proNGF have distinct signalling properties, and whether the reported differences in their activities are qualitative (*i.e.* highly distinct), or purely quantitative.

To this aim, in this paper we have undertaken a gene expression profiling study, aimed at analyzing to what extent proNGF and NGF activate different transcriptional programs in the NGF responsive cell line PC12, which expresses the full complement of NGF and proNGF receptors. PC12 cells were cultured for short times with equimolar amounts of recombinant mouse NGF (hereon simply called NGF) or two forms of recombinant mouse proNGF, wild-type, or furin-cleavage resistant (hereon simply called proNGF-WT or proNGF-KR). The gene expression changes in response to the different treatments were investigated by microarray analysis.

The results show unequivocally that, at this relatively short time scale, NGF and proNGF regulate the expression of significantly different sets of mRNAs.

## Results

### 1. proNGF- versus NGF-regulated gene expression in PC12 cells: experimental design and validation studies

The experiment was designed to verify to what extent NGF and proNGF activate different signalling pathways, thereby regulating distinct sets of mRNAs, in the NGF responsive PC12 cells, an extensively used model to study NGF-induced differentiation [31]. These cells express the full complement of NGF and proNGF receptors (TrkA, p75NTR [32] and sortilin - Figure S1). The initial signalling cascades triggered by NGF stimulation and the patterns of NGF regulated mRNAs have been well characterized in PC12 cells. On the other hand, nothing is known, so far, about how proNGF influences gene expression, in this or in other cellular systems.

The bioactivity of the recombinant NGF and proNGF proteins was verified by incubating naïve PC12 cells with equimolar amounts of NGF and the short form of proNGF, both in its wild-type (proNGF-WT) and in its furin-resistant form (proNGF-KR). The proNGF-WT and proNGF-KR proteins (Figure 1A) were expressed in *E.coli* and purified, as described in the Methods Section. The mature NGF was derived from proNGF-WT by controlled trypsin proteolysis *in vitro* [14].

We first verified through a standard PC12 cells bioassay (50 ng/mL of NGF and 100 ng/mL of the short form of proNGF, both WT and KR) that the recombinant proteins used for the experiments are able to induce an equivalent extent of morphological differentiation after 72 hours exposure (Figure S2), and are therefore active, in the frame of our experiment. PC12 cells treated with proNGF -WT and –KR had a normal morphology and not apparent sign of apoptotic cells was observed. Indeed, it has been reported that after long time exposure, both NGF and proNGF induce neurite sprouting in PC12 cells [14], [33].

The final goal of the present work was to specifically evaluate cellular responses to NGF and proNGF separately, in terms of early transcriptional expression profiles. Therefore, we focussed on a short-term exposure of naïve PC12 cells to NGF or proNGF (1 h and 4 h). Sub-saturating concentrations of the proteins were used (namely 10 ng/mL for NGF and 20 ng/mL for the short form of proNGF, both WT and KR), in order to isolate the specific early cellular responses.

**Figure 2:**
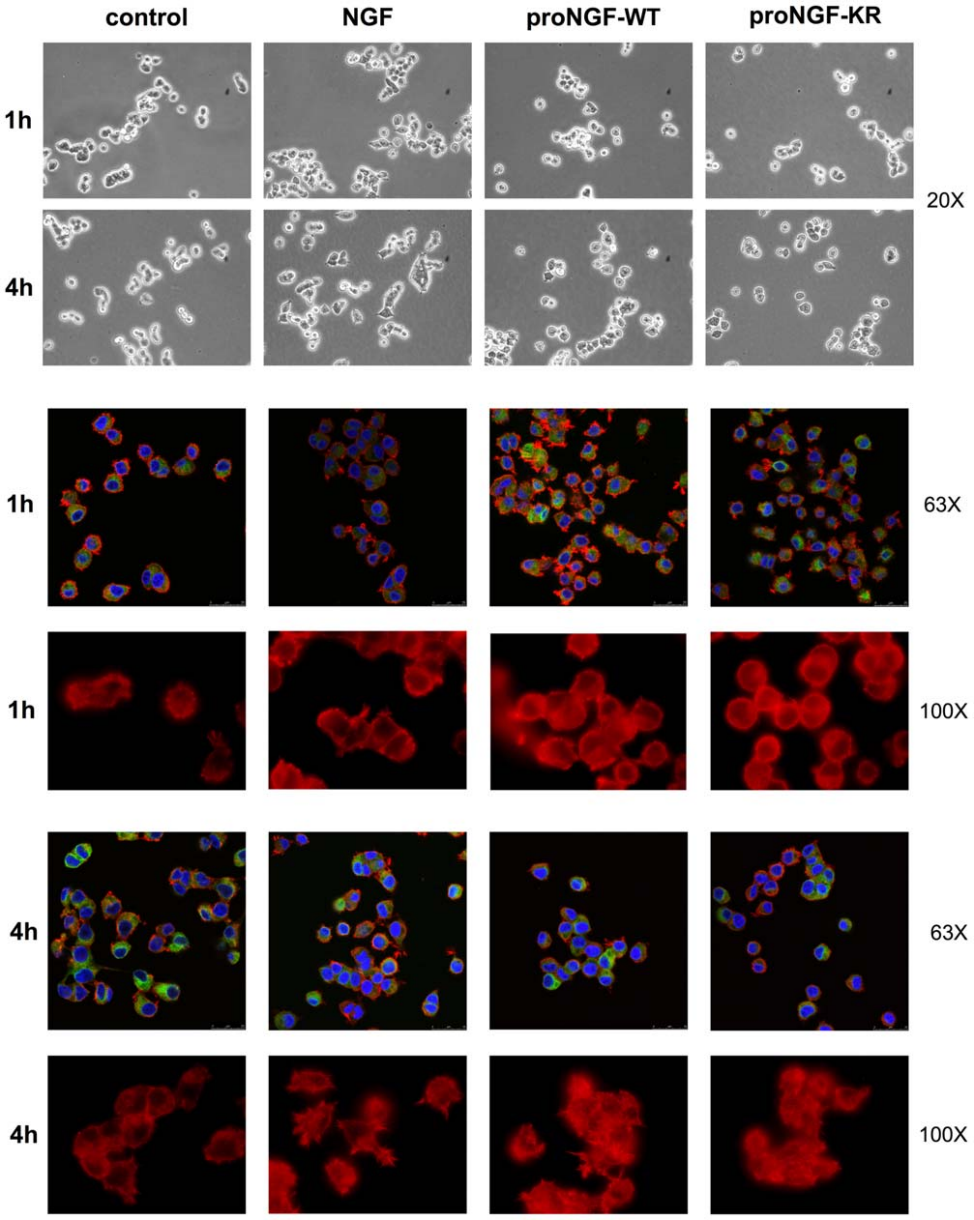
PC12 cells treated for 1 h or 4 h with equimolar amounts of the recombinant neurotrophins. Phase contrast pictures (upper two rows, 20× magnification) of PC12 cells in culture and confocal images of triple immunofluorescence of PC12 cells (last four rows) respectively for betaIII-tubulin (green), actin (red) and DNA (blue) (63× magnification) and immunofluorescence for actin cytoskeleton (100× magnification). After 1 h or 4 h cells were fixed and stained with anti-betaIII tubulin antibody, Alexa 594 phalloidin to visualize filamentous actin and DAPI for nuclear staining. - first column: control cells with no addition. - second column: 10 ng/mL of NGF. - third column: 20 ng/mL of proNGF WT. - fourth column: 20 ng/mL of proNGF KR.

We assessed the morphology of the PC12 cells in response to the NGF and proNGF 1 h and 4 h treatments. A macroscopic evaluation of the cells upon treatment with the neurotrophins (Figure 2) does not show any significant difference at the early time points used for the microarray study. Specifically, we cannot identify any difference at 1 h, while at 4 h NGF-treated cultures start showing a frequent incipient differentiated morphology, which only in rare cells is observed in both proNGF treated cultures.

The ability of this PC12 cells clone to undergo apoptosis upon treatment with proNGF was tested. In particular, cells were treated with the described amounts of neurotrophins (namely 10 ng/mL for NGF and 20 ng/mL for proNGF, both WT and KR) and apoptosis was evaluated at both 1 h and 4 h by means of TUNEL method (Terminal deoxynucleotidyl transferase dUTP nick end labeling) using the ApopTag detection Kit. We could not find any significant difference in the percentage of apoptosis induced by either proNGF-WT or –KR, when compared to the NGF treatment or the control (data not shown).

The stability of the recombinant proNGF proteins, over the same time scale of the microarray experiment, was assessed in the culture conditions of the neurotrophin treatment of PC12 cells, in order to compare the extent of processing of the wild-type and furin-resistant proNGF proteins.

A known (and measurable) amount of NGF, proNGF-WT and -KR was spiked into PC12 conditioned medium and incubated in the same conditions of temperature and time used for the cells' treatment (see Materials and Methods). The extent of proNGF degradation (the ratio between intact proNGF and the NGF produced by proNGF processing) was evaluated by Western blot analysis (Figure 1B) and densitometric analysis of the bands corresponding to those of proNGF and NGF originating from the proNGF proteolysis (Figure 1C).

As shown in Figure 1B and 1C, the proNGF samples are not cleaved upon incubation in PBS buffer, nor in fresh culture medium upon the longer incubation (4 h). As expected, incubation of proNGF samples for 1 h and 4 h in the corresponding PC12 conditioned medium yields to their partial degradation (Figure 1B). There are no substantial differences in the amount of NGF released from both proteins, at the two time points, as seen in Figure 1C.

Therefore, we conclude that while the KR mutation does not completely impair proNGF processing, due to extracellular proteases, besides intracellular furin, present in the PC12 conditioned medium [8], [9], the cleavage of proNGF-KR could be different than that of wild-type proNGF. Indeed, the kinetic of processing of the WT and the KR mutant are not easily measurable. Therefore, we cannot exclude that there might be different cleavage kinetics of the two proteins *in vivo*. The kinetics would account for a difference in the NGF/proNGF ratio in the system in the two cases, during the proteolysis progression, at different time points.

**Figure 3:**
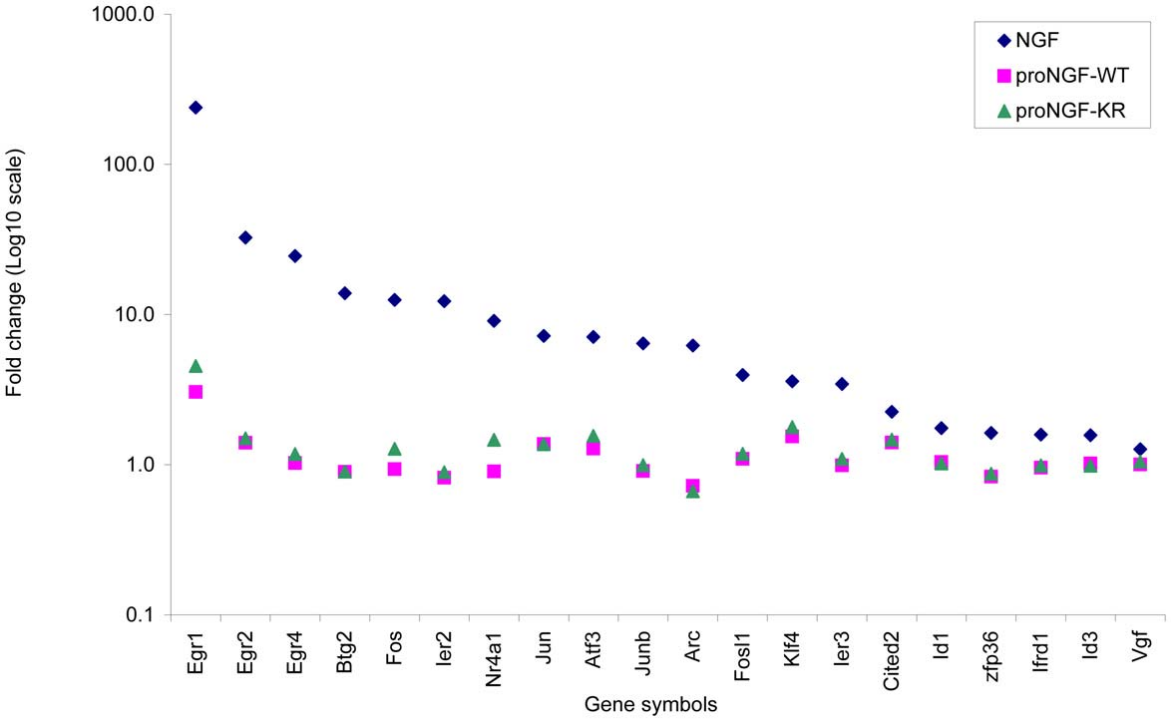
Relative expression data of typical early NGF-responsive genes, 1 hour after NGF, proNGF-WT, proNGF-KR treatments. The gene symbols refer to the following gene names: Egr1 (also known as Tis8) = early growth response 1; Egr2 = early growth response 2; Egr4 = early growth response 4; Btg2 (also known as Pc3) = B-cell translocation gene 2, anti-proliferative; Fos = FBJ murine osteosarcoma viral oncogene homolog; Ier2 = immediate early response 2; Nr4a1 (also known as Tis1) = nuclear receptor subfamily 4, group A, member 1; Jun = Jun oncogene; Atf3 = activating transcription factor 3; Junb = Jun-B oncogene; Arc = activity-regulated cytoskeleton-associated protein; Fosl1 = fos-like antigen 1; Klf4 = Kruppel-like factor 4 (gut); Ier3 = immediate early response 3; Cited2 = Cbp/p300-interacting transactivator, with Glu/Asp-rich carboxy-terminal domain, 2; Id1 = Inhibitor of DNA binding 1, helix-loop-helix protein (splice variation); zfp36 (also known as Tis11) = zinc finger protein 36; Ifrd1 (also known as Pc4) = interferon-related developmental regulator 1; Id3 = inhibitor of DNA binding 3; Vgf = VGF nerve growth factor inducible.

The transcriptional profile regulated by NGF was analyzed first, in order to set a comparison with published data [34]–[48], confirming the well established activation of immediate early gene after NGF treatment of PC12 cells [37], [47], [49], [50]. Figure 3 reports the analysis for immediate early genes and for other genes known to be induced early by NGF. Many of these genes encode possible transcription factors, and thus may play roles in the initiation and regulation of subsequent responses to NGF (for review, [38]).

It is noteworthy that none of these immediate early genes is activated to any significant extent by either proNGF-WT or proNGF-KR (Figure 3), suggesting already from the analysis of this first set of genes that NGF and proNGF may activate distinct transcriptional programs.

### 2. Distinct mRNA expression patterns are regulated by NGF and proNGF

The differential activation of immediate early genes by NGF *versus* proNGF (Figure 3) suggests that their transcriptional responses may be significantly different. Therefore, the overall statistics of the whole dataset of differentially expressed genes were evaluated. This analysis showed that NGF and proNGF regulate distinct mRNA sets in PC12 cells (Table S1), over the time scale investigated.

**Figure 4:**
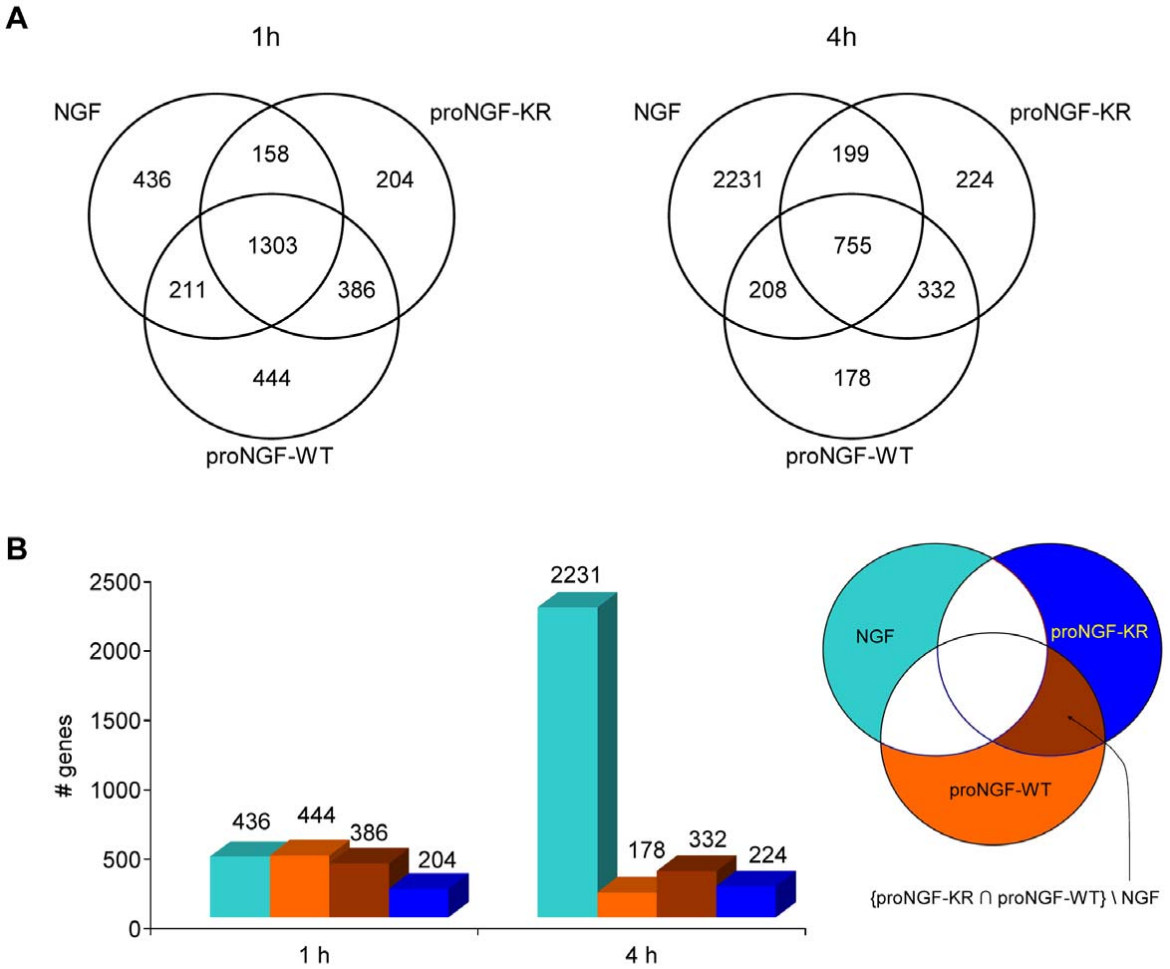
Overall statistics of differentially expressed genes. We selected genes with a fold change larger than 1.2 or lower than 1/1.2 in the linear scale. Only genes with a gene symbol annotation were considered. Differential genes counts are shown for each of the three different treatments, either 10 ng/ml NGF or 20 ng/ml of proNGF-WT or -KR, at the two selected time points (1 h and 4 h). (A) Counts of differential genes in the different regions of the Venn diagram referring to the three treatments; all the seven gene sets are disjoint, at each time point, with no element in common. (B) Counts of differential genes in four highlighted gene sets: color of bars correspond to the regions in the Venn Diagrams on the right.

Figure 4A illustrates the overall counts of the differential genes. Among the different intersection areas of the Venn diagram, we focussed our analysis on the coloured regions shown in Figure 4B, highlighting the specific proNGF fingerprinting compared to the NGF one. Genes activated exclusively by NGF are found in the cyan region; genes activated by proNGF WT or by proNGF KR are represented in orange and blue, respectively; the brown region represents the overlap of the genes activated by proNGF WT and KR, but not by NGF. We refer to the brown region as the “pure proNGF” activated genes set. The “pure proNGF” set identifies, therefore, the genes most likely to be affected exclusively by the proNGF component of the neurotrophin mixture, and provides therefore a fingerprint of proNGF activity on a given cell. It is interesting to notice that in this “pure proNGF” intersection set, all the genes have the same trend in both the proNGF-WT and -KR preparation, that is either both up-regulated or both down-regulated (Table S1).

At 1 h, the total number of mRNAs exclusively regulated by NGF (N = 436, “pure NGF” cyan colour in Figure 4B) is very similar to the total number of mRNAs regulated by proNGF-WT (N = 444 orange colour in Figure 4B), while proNGF-KR regulates a lower number of mRNAs (N = 204, blue colour in Figure 4B). The picture changes completely at 4 hours, when the total number of differentially expressed genes is very different for the proNGF or NGF treated cultures, showing that the number of genes up- or down-regulated by proNGF-WT (N = 178, colour orange in Figure 4B) or the pro-NGF-KR (N = 224, colour blue in Figure 4B) is about ten times lower than the number of mRNAs differentially regulated by NGF (N = 2231, colour cyan in Figure 4B). The number of the differentially genes of the “pure proNGF” genes is unchanged at 1 and 4 hours.

In the case of NGF treated cells, the increase in the amount of differential genes from 1 h to 4 h time is a result of the well known early activation of transcription factors following NGF exposure. proNGF does not share this property of NGF, showing a more restricted response at 4 hours.

**Figure 5:**
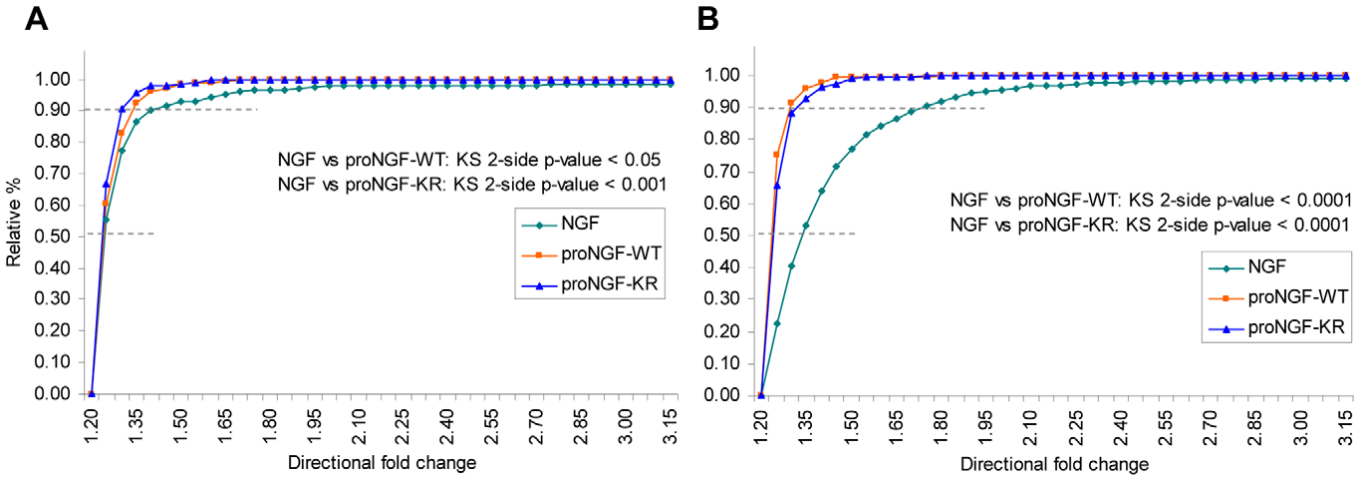
Distribution of directional fold changes. Cumulative distribution (normalized to 100%) of directional fold changes values (linear scale) for differentially expressed genes, in the three disjoint sets NGF, proNGF-WT, proNGF-KR (see Figure 4B), at 1 hour (A) and 4 hour time (B). Genes were selected by one-way ANOVA (p<0.05), assuming not equal variance. The fold change values (fc) for down-regulated probes, by definition <1.0, were converted into values >1.0 as 1/fc. The distributions of fold change, NGF *vs* proNGF-WT and NGF *vs* proNGF-KR, were clearly distinct and were compared using the 2-side Kolmogorov-Smirnov test for two samples. The fold change values at 50% of the distribution at 1 h for (NGF, proNGF-WT, proNGF-KR) are respectively (1.25, 1.23, 1.23) while at 4 h they are (1.34, 1.22, 1.23). The foldchange values at 90% of the distribution at 1 h for (NGF, proNGF-WT, proNGF-KR) are respectively (1.40, 1.32, 1.30) while at 4 h they are (1.74, 1.29, 1.32).

The differential regulation of gene expression in PC12 by NGF and proNGF was further analyzed by comparing the corresponding distributions of relative frequencies of “fold variation” ratio (NGF or proNGF treated PC12/naïve PC12) for differentially expressed mRNAs (Figure 5). As shown in the Figure 5, the overall cumulative distribution for NGF regulated genes is shifted towards higher fold variation values than the overall distribution for proNGF-WT and proNGF-KR regulated genes. The fold change values at 90% of the cumulative distribution at 1 h for NGF, proNGF-WT and proNGF-KR are respectively 1.40, 1.32 and 1.30, while at 4 h they are 1.74, 1.29 and 1.32 respectively. The fold change distributions were compared using the 2-tails Kolmogorov-Smirnov test for two samples and were found to be significantly different. Thus, NGF exerts a much more potent and widespread regulation of mRNA expression than proNGF, both in terms of number of regulated genes and of fold variation.

### 3. Functional analysis of NGF and proNGF regulated genes

The microarray datasets were functionally analyzed by bioinformatics tools, to learn more about the nature of differentially expressed genes and about specific mechanisms or pathways significantly modulated in the different experimental groups. To this aim, differentially expressed genes for each treatment and time point were grouped into functional categories. To this aim, 208 differentially expressed genes for each treatment and time point were grouped into 209 functional categories, by the Panther Ontology tool. As a first step, using the Panther onthology tool, 8 disjoint lists of statistically significant Panther Biological Processes were obtained (Table S2), one for each treatment (NGF, proNGF-WT, proNGF-KR and the intersection) and time point (1 and 4 hours), using the whole filtered normalized transcriptomic dataset (see Materials and Methods). Starting from these Panther categories, a more detailed functional analysis was performed, by investigating, out of the whole normalized dataset, only the differentially expressed genes. Therefore, as a second step, 8 sets of differentially expressed genes were compiled, showing fold-change values larger than 1.2 or lower than 1/1.2 in the linear scale (see Materials and Methods): these genes were further filtered by selecting those belonging to statistically significant Panther Biological Processes, and eventually classified into the categories summarized in Table 1.

**Table 1:** Main functional categories of differentially expressed genes belonging to the four analyzed set regions shown in the Venn diagram of Figure 4B.

NGF 1 h	NGF 4 h	proNGF-WT 1 h	proNGF-WT 4 h	proNGF-KR 1 h	proNGF-KR 4 h	proNGF 1 h	proNGF 4 h
cell cycle	cell cycle	cell cycle	cell cycle		cell cycle	cell cycle	cell cycle
intracellular trafficking / synaptic activity	intracellular trafficking / synaptic activity	intracellular trafficking / synaptic activity	intracellular trafficking / synaptic activity		intracellular trafficking / synaptic activity	intracellular trafficking / synaptic activity	intracellular trafficking / synaptic activity
DNA repair	DNA repair	DNA repair		DNA repair	DNA repair	DNA repair	
ionic trasport	ionic trasport	ion transport				ion transport	ion transport
stress response						stress response	stress response
	kinases and phosphatases					kinases and phosphatases	
	carbohydrate metabolism					carbohydrate metabolism	
transcription factor	transcription factor					transcription factor	
development				development			
		lipid metabolism				lipid metabolism	
	DNA replication						
	RNA processing						
		Embryonic development					
						structural proteins	
							cell adhesion
							cell migration
						chaperones	
						receptors	

Main functional categories of differentially expressed genes belonging to the four analyzed set regions shown in the Venn diagram of Figure 4B: NGF\{proNGF-WT U proNGF-KR} (light blue in Figure 4B, NGF in Table 1), proNGF-WT\{NGF U proNGF-KR} (orange in Figure 4B, proNGF-WT in Table 1), proNGF-KR\{NGF U proNGF-WT} (blue in Figure 4B, proNGF-KR in Table 1), {proNGF-KR ∩ proNGF-WT}\NGF (brown in Figure 4B, proNGF in Table 1).

Among all the functional categories listed in Table 1, the following ones were analyzed in details: the transcription factor family, well represented only in the NGF treatment dataset, the lipid metabolism one, specific for the “pure proNGF” set, and the cell cycle and DNA repair families, that are common between NGF and proNGF specific sets.

**Figure 6:**
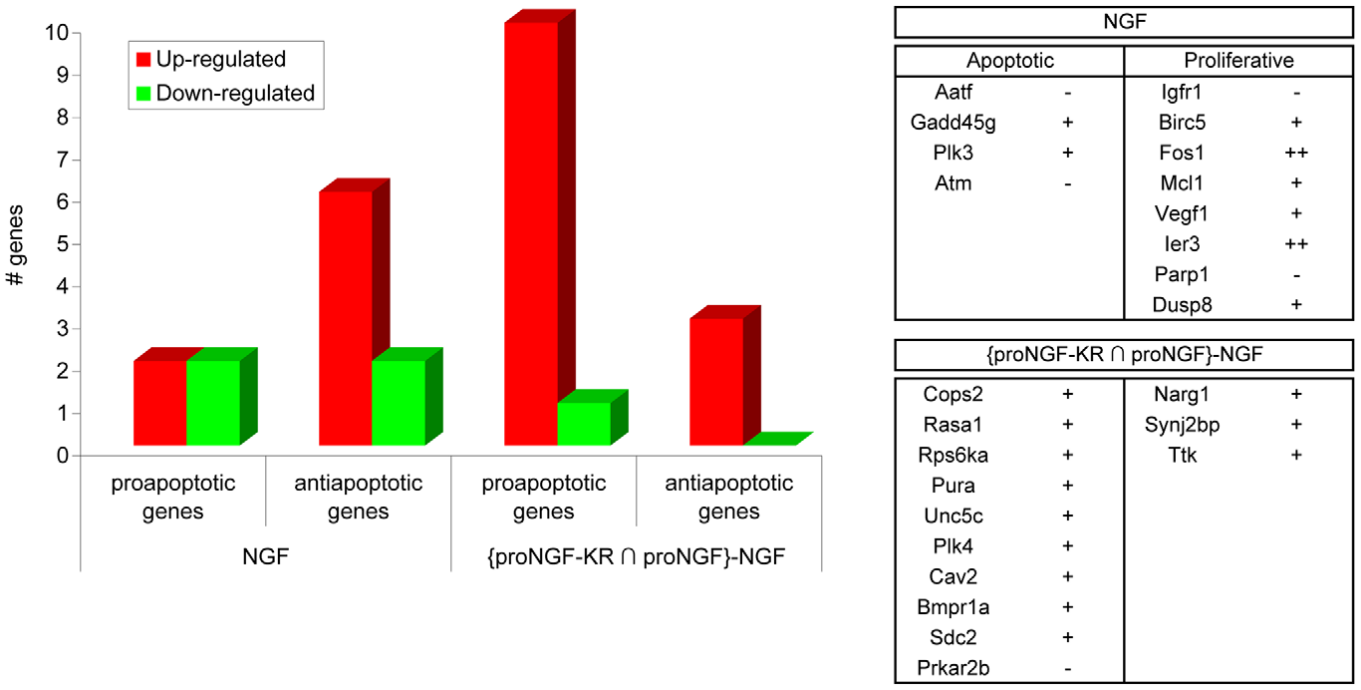
Summary of regulated cell-cycle genes. Summary counts of up-regulated and down-regulated cell cycle genes with a clear involvement in apoptotic and proliferative processes, for the NGF treatment and for the intersection between proNGF-WT and –KR at 1 h treatments. In the tables on the right, the names of the counted genes with the corresponding up-regulated (+) or down-regulated (−) trend.

The cell cycle category is well represented, both in the NGF and in the proNGF treatments. Analyzing in detail the genes included in this category for the NGF specific set and the proNGFs intersection set, after 1 h treatment, two distinct trends can be highlighted (Figure 6). We observe indeed that, after 1 h treatment, while NGF activates the expression of a mitotic and anti-apoptotic outcome mRNAs, proNGFs activates pro-apoptotic genes, including tumour suppressor mRNAs. It is noteworthy that the “proliferative signature” is conserved for NGF, also at 4 hours of treatment, while the proapoptotic signature is not maintained for proNGFs transcriptional response at 4 hours. This could be due to a contribution of the fraction of mature NGF derived from the cleavage of the precursor, a contribution that increases with incubation time.

The differential regulation of genes coding for transcription factors represents another major distinction signature of NGF versus proNGF in PC12 cells. A summary count of the transcription factors differentially expressed in this system by NGF and proNGF is reported in Table 2, that shows a reduced number of transcription factor genes represented in the proNGF intersection set, compared to the NGF treatment set at 1 hour. The number of the differentially expressed transcription factor genes in the “pure proNGF” or NGF treated cells is strikingly different at 4 h, being much lower in the proNGF group, pointing out a specific fingerprinting of the proNGF.

**Table 2:** Summary counts of the main up- and down-regulated transcription factors genes.

	NGF\{proNGF-WT U proNGF-KR}		(proNGF-WT ∩ proNGF-KR)\NGF	
	Up ↑	Down ↓	Up ↑	Down ↓
***1 h***	30	0	13	0
***4 h***	114	13	0	0

Summary counts of up- and down-regulated transcription factors genes with a fold change larger than 1.2 or lower than 1/1.2 in the linear scale, for the NGF treatment and for the intersection between proNGF-WT and -KR. The headers correspond to the regions in the Venn diagram of Figure 4B and the gene sets, at each time point, are disjoint. The transcription factors belong to the sets in Figure 4B {NGF\{proNGF-WT U proNGF-KR}} (left side – corresponding to the blue in Figure 4B) and {{proNGF-KR ∩ proNGF-WT}\NGF} (right side – corresponding to the brown in Figure 4B).

Table 3 describes the analysis of the main functional categories of genes whose expression levels correlate with those of the transcription factors modulated by the NGF or proNGF. This kind of analysis reconstructs a snapshot of the general status of the cell. ProNGF signalling indicates an early involvement of mitochondrial and metabolic genes, while NGF confirms the induction of the transcription response, gene expression and angiogenesis related genes. The picture of the cell treated with NGF is characterised by an increased metabolism of the cell preparing itself to progress toward the cell cycle division, while proNGF treated cell could be less active.

**Table 3:** Functional analysis of genes induced by the transcription factors modulated by NGF and proNGF after 1 h of treatment.

	Functional cluster	Geometric mean of cluster terms p-values
**NGF**	regulation of gene expression	2.02E-06
	regulation of transcription	1.05E-05
	regulation of angiogenesis	1.58E-03
**proNGF**	mitochondrial membrane	3.09E-04
	RNA transport	1.90E-02
	regulation of metabolic process	2.61E-02

Main DAVID functional clusters of gene lists obtained by computing the standard correlation of transcription factors after 1 h, listed in Table 2, with the expression values of the whole set of data and selecting only the genes with absolute correlation value >0.90. The transcription factors belong to the sets in Figure 4B {NGF\{proNGF-WT U proNGF-KR}} (light blue in Figure 4B, NGF in Table 3) and {{proNGF-KR ∩ proNGF-WT}\NGF} (brown in Figure 4B, proNGF in Table 3).

Other differences between NGF and proNGF signalling reside in DNA replication and chromatin remodelling gene families, where several genes involved in DNA repair are differentially expressed after exposure to either NGF or proNGF (-WT or -KR), but in opposite directions. NGF at 4 h mainly down-regulates DNA repair genes, such as DNA polymerase subtype, primarily involved in repair and other related enzymes. Instead, 1 h of treatment with proNGF (either -WT or -KR) up-regulates DNA repair genes, both specific DNA polymerase involved in repair mechanisms and other single-strand and double-strand binding proteins that repair DNA breaks by recombination.

As for the “pure proNGF” set, many genes involved in carbohydrate- as well as in lipid-metabolism were found to be significantly down-regulated. Interestingly, it has been recently shown [51] that proNGF is modified by non-enzymatic glycation and lipidation in AD (Table 1), although we cannot directly compare these modifications with the regulation by proNGF of carbohydrate and lipid post-translationally modifying enzymes, highlighted in PC12 cells.

We then analyzed the genes specific for proNGF-WT and proNGF-KR, represented in the Venn diagrams by the orange and blue colours, respectively. The two proNGF-WT and -KR specific datasets contain different number of genes and different gene classes, suggesting that these two proteins behave somewhat differently. Since the two proNGF –WT and –KR proteins have been widely used interchangeably [8], [52] and functional differences among them have not been reported, we ascribe the transcriptional differences highlighted in this analysis to their differential processing during the incubation with PC12 cells, and hence to a distinct contribution by mature NGF in the two conditions.

The results underscore the importance of the relative amount of NGF *versus* proNGF in the biological outcome. It appears therefore critical, when taking into account the signalling fingerprinting of proNGF, to consider both the amount of mature and precursor protein, particularly for *in vivo* situations.

One gene family significantly modulated in the two treatments at 4 h is linked to synaptic functions and activity (genes involved in vescicular transport, ion channels, protein trafficking), although in the case of proNGF-WT, these genes are all down-regulated, while in the case of proNGF-KR they do not have a homogeneous trend, being partly up- and partly down-regulated.

Finally, the expression trend of specific genes, known to be linked to NGF and proNGF activity, were sought and analyzed in the different datasets.

We could identify furin to be down-regulated in “pure proNGF” set, suggesting a feedback regulation loop possibly fine-tuning and reinforcing proNGF activity, by reducing its metabolism. Significantly, in the proNGF-WT at 1 h, the TrkA receptor gene is down regulated, further suggesting a feedback effect of proNGF-WT, leading to a reduced efficacy of signal transduction mediated by TrkA receptor.

Despite the evidence of the cross-talk between the p75^NTR^ and sortilin in the cell death induced by proNGF, we could not find a modulation in these receptors' genes in the proNGF treatments. However, this agrees well with the finding that the protein levels of p75^NTR^ and sortilin are unaffected in neurodegeneration states [25], [26].

Another gene recently discovered to be regulated by proNGF in rat CNS neurons, the phosphatase and tensin homolog deleted on chromosome 10 gene pTen [53], is not differentially expressed in our system.

## Discussion

The cellular response to NGF has been extensively studied in the PC12 cell line, both in terms of the cellular phenotype, of signalling, and, more recently, of the transcriptional profiles [45], [54]. In light of recent studies pointing to an independent and distinct biological role for the NGF precursor protein proNGF, particularly relevant in neurodegeneration, we investigated the properties of proNGF signalling by gene expression microarray, since very little is known in this respect, for proNGF.

The aim of the experiment was to exploit transcriptional regulation as a “signalling signature” to address the question whether NGF and proNGF show only quantitative or also qualitative differences in their respective transcriptional activation programs.

The present is therefore the first study, aimed at an overall comparison of the genes induced in PC12 cells upon treatment with mature NGF or its precursor.

In order to isolate as much as possible the effects of a “pure” proNGF system, we treated the cells either with proNGF-WT or with the furin resistant mutant proNGF-KR. A limited partial processing of the proteins by other extracellular protease still occurs, as also demonstrated in the literature [8], [9]. Therefore we concentrated on the early response in the system (1 and 4 hours), when the processing of the proNGF proteins is lower. Using this approach, we were able to conclude that NGF and proNGF activate distinct transcriptional programs and to identify a specific proNGF transcription signature, distinct from NGF.

Our results clearly show that NGF and proNGF signalling mediate distinct mRNA expression patterns, not only in terms of total number of modulated genes (a higher number for NGF than for the proNGFs – See Figure 4), but also in terms of gene families (see Table 2).

The functional analysis of NGF-induced transcriptional data allowed us, at first, to confirm previously published studies on NGF-induced microarray profiles in PC12 cells. Indeed, we observed that transcription factors and gene expression related processes are heavily induced by NGF.

We then analyzed the system by taking into account certain subsets of differentially expressed genes. In particular, we focussed on the intersection set genes induced both by proNGF-WT and proNGF-KR, that we called the “pure proNGF” subset. We compared this identified group of genes, with those activated by NGF and with those activated by either proNGF-WT or proNGF-KR selectively.

In general, we observed in the proNGF transcriptional activity the absence of certain gene families heavily activated by NGF. We could identify certain gene families mainly activated by the “pure proNGF”. Most significantly, proNGF was shown to induce genes connected to carbohydrate and lipid metabolism.

Although in a different context, it has been recently shown [51] that proNGF is modified by non-enzymatic glycation and lipidation in AD, therefore this kind of modifications could be interpreted as a specific signature of the protein. It is remarkable that the modulation of the lipid metabolism, and of genes of the cholesterol biosynthesis among these, is a specific signature for the proNGF treatment. Indeed, it has been shown that cholesterol biosynthesis is connected on one side to the p75^NTR^-mediated signalling and apoptosis [55]–[57], and on the other side to the progression of AD [58], [59]. Given the proposed role of proNGF in p75^NTR^-mediated apoptosis [8] and the unbalance of the proNGF/NGF ratio in AD [18], [27], [60], further analysis will be required to evaluate the importance of this pathway in the specific biological outcome of proNGF in cellular systems and *in vivo*.

A further discriminating category between NGF and proNGF is the cell cycle family, encompassing mainly pro-proliferative genes in the case of NGF and pro-apoptotic genes in the case of proNGF at 1 h of treatment. Other mRNA families distinctly regulated involve DNA replication and chromatin remodelling, which are differentially expressed after exposure to either NGF or proNGF, but usually in opposite directions, which leads to suggest a differential effect of the two neurotrophin forms even on common pathways.

Particularly notable is the difference in the regulation of mRNAs coding for transcription factors. In particular, proNGF was found to modulate a smaller number of transcription factor genes compared to NGF and the treatment of PC12 cells with NGF or proNGF appears to have a completely different effect on the cellular response. While in the case of NGF, the modulated transcription factors are connected with a regenerative/differentiative trend, those modulated by proNGF are more connected with a less proliferative cell.

From our analysis, we suggest that the relative ratio of NGF *versus* proNGF is critical for the downstream transcriptional signalling. In fact, we observe that there is a significant number of genes selectively modulated by proNGF-WT or proNGF-KR, and that for each of the two subsystems, the genes overlapping with those of NGF in the two cases are also different. This observation well fit with our hypothesis that the kinetic of interconversion of proNGF-WT and –KR into NGF is likely to be different, due to the removal of one dibasic aminoacid site in the proNGF-KR. The consequence of a different proteolytic kinetic is that the PC12 cells system is exposed to a progressively different NGF/proNGF protein ratio, in the time windows considered. This could account for the difference between the two datasets. The relative ratio of NGF/proNGF is surely important for the biological response of the cellular treatment with the neurotrophins.

The regulation of the NGF/proNGF ratio *in vivo* might therefore have profound consequences, further underscoring the crucial importance of determining this parameter in different *in vivo* situations.

These results fit well with data in the literature [30], showing how the biological outcome of proNGF is finely tuned by the amount of protein, as well as by the relative ratios of the involved receptors. Our results help also explaining why proNGF is able to induce distinct biological effects in different cellular systems and different biological conditions [8], [17], [29], [61]. A strong unbalance towards “pure” NGF is unlikely to be found *in vivo*, where the co-existence of the mature and precursor neurotrophins has been widely observed. Moreover, in pathological conditions, such as brain injury or Alzheimer's disease, the equilibrium between synthesis and cleavage of proNGF was demonstrated to be in favour of the precursor form [18], [52]. Therefore, the life/death effects induced by NGF and proNGF appear to be strictly related to their relative ratio *in vivo*. Indeed, being the two neurotrophin forms able to activate different transcriptional programs, we suggest that the biological effects exerted *in vivo* are a result of a complex balance between their specific signalling and transcriptional programs.

In conclusion, we report herein the first characterization of the differential transcriptional signature of proNGF *versus* NGF, in PC12 cells. From our data, we confirm that the mature and the precursor proteins are biologically different, and show a different transcriptional signature. These results open the path for subsequent more detailed studies of the distinct transcriptional pathways activated by NGF and proNGF, in order to better characterize their different biological activity *in vivo*.

## Materials and Methods

### Cloning and expression of mouse short proNGF (proNGF)

The cDNA sequence of the short form of proNGF, corresponds to bp 348 to 1010 (NCBI entry M35075); from aminoacid −103 to aminoacid +118 (Figure 1A). proNGF was cloned and expressed in *E. coli* according to the protocols described in [14].

The mutant protein proNGF-KR carries a change from aminoacids KR to AA at position −2, −1 (Figure 1A), which destroys the cleavage site by furin protease [52]. The mutant was obtained by site-directed mutagenesis by Stratagene Kit. The correct cDNAs were subcloned into the pET11a vector (Novagen). The protein was expressed following the same protocol described for wild-type proNGF [14]. NGF was obtained from proNGF, by “*in vitro*” proteolytic cleavage with trypsin [14].

### Receptors characterization in the PC12 SB subclone

Rat pheochromocytoma PC12 cells [31] (PC12 SB subclone) were maintained with RPMI 1640 Medium (Invitrogen) and grown as monolayer cultures on Falcon dishes, supplemented with 10% Horse Serum (Invitrogen) and 5% Foetal Calf Serum (Invitrogen), in a humidified atmosphere at 37°C and 5% CO_2_.

Cell dishes were washed with PBS, incubated on ice with lysis buffer (50 mM Tris pH 7.4, 150 mM NaCl, 1% Triton X-100, 0.1% SDS, 10 mM EDTA), then scraped and centrifuged. Protein concentration of the supernatant was evaluated by Bradford Assay (Sigma). 60 µg of the total protein lysate was loaded onto a Criterion™ XT Bis-Tris Gel, 4–12% (BioRad) to perform Western Blotting. The membrane was cut and challenged with anti TrkA (Chemicon), anti Sortilin (R&D System) and anti p75NTR (Alomone Labs), according by the manufacturer's protocol, and then with the proper secondary antibodies, HRP conjugated, at 1∶7000 (all by Jackson Lab).

### PC12 cells treatment and differentiation bioassay

Rat pheochromocytoma PC12 cells [31] (PC12 SB subclone, kindly provided by Maurizia Caruso, Consiglio Nazionale delle Ricerche, INMM, Rome, Italy) were maintained with RPMI 1640 Medium (Invitrogen) and grown as monolayer cultures on Falcon dishes, supplemented with 10% Horse Serum (Invitrogen) and 5% Foetal Calf Serum (Invitrogen), in a humidified atmosphere at 37°C and 5% CO_2_. For differentiation, PC12 cells were plated at a concentration of 10^6^/dish (100 mm plates, BD Falcon) and kept in culture for 12 hours. Cells were treated with 10 ng/ml NGF, 20 ng/ml of wild type (WT) proNGF or 20 ng/ml of furin-cleavage resistant proNGF-KR.

After 72 hours, the medium was changed and free medium+proteins was supplied. Following different incubation times, light microscope pictures were taken of the living differentiating cells and neurite extension was evaluated.

For microarray mRNA expression analysis, half volume of medium was replaced with fresh medium and the naïve PC12 cells were treated for 1 h or 4 h with 20 ng/mL of proNGF or proNGF-KR or 10 ng/mL NGF. A negative control with no neurotrophin addition was performed.

In order to evaluate cells morphology in the same experimental conditions used for RNA extraction, a triple immunofluorescence was performed on PC12 cells exposed to analogous treatments (20 ng/mL of proNGF or proNGF-KR or 10 ng/mL NGF). After 1 h or 4 h cells were fixed [62] and stained with anti-beta III tubulin antibody (Covance, 1∶250 dilution), Alexa 594 phalloidin (Invitrogen, 1∶40 dilution), to visualize filamentous actin, and DAPI for nuclear staining. Coverslips were mounted using Vectashield (Vector) mounting medium. Stained cells were analyzed by confocal laser scanning microscopy on Leika microscope.

The apoptosis in PC12 cells was evaluated with the TUNEL method (Terminal deoxynucleotidyl transferase dUTP nick end labeling) using the ApopTag detection Kit (Merck Millipore), according to manufacturer's instructions.

### Determination of proNGF stability

The processing of the proNGF proteins in the PC12 cells culture was evaluated by a spiking experiment. The amount of neurotrophins used in the PC12 cells treatment for the microarray experiments is too low to be detected in a direct Western Blot analysis, and does not allow to quantify the precise percentage of NGF cleaved from the two forms of the precursor during the incubation. To this aim, a sufficient amount of the recombinant proteins was spiked into the conditioned medium (CM) of treated PC12 cells, in the same conditions of temperature and time used for the microarray experiment. In details, 1 µg of recombinant proNGF-WT, proNGF-KR or NGF was spiked into 50 µL of 1 h the conditioned medium (CM) or 4 h CM of treated PC12 cells, in the same conditions of temperature and time used for the microarray experiment.

The spiked medium was incubated at 37°C for 1 h or 4 h and then 20 µL/sample were run on SDS-PAGE for Western blotting analysis with an anti-NGF antibody (Jackson Lab).

PC12 cells were washed, fresh medium was added and kept for 1 h or 4 h (1 h CM or 4 h CM). Primary antibody: anti-NGF M13 (Santa Cruz), at a 1∶200 concentration, 16 hours at 4°C. Secondary antibody: Goat Anti-Rabbit, HRP conjugated (Jackson Lab), 1∶7000, 1 hour at room temperature.

The ratio between the intact proNGF and the NGF produced by processing from input proNGF (degradation ratio) was determined by a densitometric analysis of the bands in each lane, performed with the Kodak digital imager.

### RNA isolation

RNA was isolated from four different PC12 cell cultures, PC12 cells treated with NGF, wild type proNGF (proNGF-WT) or furin resistant proNGF (proNGF-KR) or untreated cells. Neurotrophin treated cell cultures were sampled at two different incubation times (1 and 4 hours). Two biological replicates were used for each time point and treatment.

PC12 cell cultures were scraped and lysed with Trizol (Invitrogen) and DNAse treated by Qiagen columns. RNA quantity was determined on a NanoDrop UV-VIS. Only samples with an absorbance ratio of 1.8<OD_260_/OD_280_<2.0 were processed further. Each sample was then quality checked for integrity using the Agilent BioAnalyzer 2100 (Agilent G2938C, RNA 6000 nano kit): samples with a RNA Integrity Number (RIN) index lower than 8.0 were discarded. 500 ng of RNA were used for each reaction. cRNA was synthesised from double-stranded cDNA during in vitro transcription with T7 RNA polymerase, labelled using Cyanine 3-CTP or Cyanine 5-CTP (Perkin Elmer) and purified (Qiagen's RNeasy mini spin columns).

### Hybridization of oligonucleotide Rat microarrays

The gene expression profiling was performed using the two-color Agilent protocol (http://www.chem.agilent.com/en-US/Products/Instruments/dnamicroarrays/Pages/default.aspx).

cRNA samples from the treated cells were labelled by Cyanine-5, while the control samples (from untreated cells) were labelled by Cyanine-3. Labelled cRNA samples (825 ng each sample) were hybridized to Agilent 4×44 k whole Rat genome oligonucleotide microarrays (G4131F) at 65°C for 17 hours using Agilent's Gene Expression Hybridization Kit. The hybridized microarrays were disassembled at room temperature in Agilent Gene Expression Wash Buffer 1. After the disassembly, the microarrays were washed in Gene expression Buffer 1 for one minute at room temperature, followed by washing with Gene Expression Wash Buffer 2 for one minute at 37°C. The microarrays were then treated with acetonitrile for one minute at room temperature.

### Scanning, feature extraction and analysis

Post-hybridization image acquisition was accomplished using the Agilent scanner G2564B, equipped with two lasers (532 nm and 635 nm) and a 48 slide auto-sampler carousel. The “extended range” scanning protocol was used, where the output of two following scannings at 10% and 100% of laser power are numerically combined. Data extraction from the images was accomplished by Agilent Feature Extraction 9.1 software, using the standard Agilent two-color gene expression extraction protocol (GE2-v4_91).

Raw data filtering was performed in Microsoft Excel using any of the following criteria to discard spots: spots with more than 5% of saturated pixel in any of the two channels, spots flagged as “not found” by the Feature Extraction software in any of the two channels, spots with a Signal/Noise ratio smaller than 3 in any of the two channels, where Signal = (median of the spot−median spot background level) and Noise is the IQR (interQuantileRange) of the median spot background. Data analysis was performed on filtered data using Agilent GeneSpring GX 7.3 and Microsoft Excel. Each array was normalized by the Lowess algorithm within GeneSpring, using 20% of data as smoothing window.

Differentially modulated gene families were identified bioinformatically from the Panther (Protein ANalysis THrough Evolutionary Relationships) database of Biological Processes (http://www.pantherdb.org/tools/genexAnalysis.jsp), using the Wilcoxon Rank-Sum Test (p-value<0.05) on two-column tables including the list of differentially expressed genes and the related fold change values. The PANTHER Classification System is a freely available web-based resource that classifies genes by their functions, using both published experimental evidence and evolutionary relationships for predictions. Genes and proteins are classified by expert curators according to: Gene families and subfamilies, Gene Ontology classes (molecular function, biological process, cellular component), PANTHER Protein Classes, Pathways. The gene lists used for this Panther analysis were obtained from the whole set of filtered and normalized data, with the following two additional criteria: genes without an official Gene Symbol were excluded from the analysis; in the case of more than one mRNA probe referring to the same gene, the most significant value (based on the largest absolute log_2_ value of fold change) was chosen.

As a result, 4 lists of statistically significant Panther categories were obtained, one for each treatment (NGF, proNGF-WT, proNGF-KR) and one for the intersection between proNGF-WT and proNGF-KR treatments, including in each list only disjoint category terms. Each of these lists was analyzed at two time points (1 and 4 hours).

Four disjoint sets of differentially expressed genes, showing directional fold change larger than 1.2 or lower than 1/1.2 in the linear scale, were compiled: one for each treatment (NGF, proNGF-WT, proNGF-KR) and one for the intersection between proNGF-WT and proNGF-KR treatments, at the two time points (1 and 4 h) (see the Venn diagram in Figure 4). Only the genes with a match in the corresponding sets of statistically significant Panther terms, based on the gene annotation in the Panther database, were eventually selected.

Transcription factors were identified by the DAVID tool [63] from the sets of differentially expressed genes; DAVID was also used for annotation of individual genes.

## Supporting Information

Figure S1 **PC12 cells treated with the recombinant neurotrophins – 72 h after treatment.** *Panel A* – 50 ng/mL of NGF. *Panel B* – 100 ng/mL of proNGF-WT. *Panel C* – 100 ng/mL of proNGF-KR. *Panel D* – Control without addition of neurotrophins.
(TIF)

Figure S2 **Receptors characterization in the PC12 SB subclone.** PC12 cells lysate was subjected to Western blotting. The antibodies for the three receptors (TrkA, p75NTR and sortilin) were used.
(TIF)

Table S1 **List of differentially expressed genes at the two selected time points, 1 and 4 hours.** Selected genes have an absolute directional fold change larger than 1.2 and an official gene symbol annotation. The colours on the top correspond to the highlighted subsets in the Venn diagram of Figure 4B: NGF\{proNGF-WT U proNGF-KR} in light blue, proNGF-WT\{NGF U proNGF-KR} in orange, proNGF-KR\{NGF U proNGF-WT} in blue, {proNGF-KR ∩ proNGF-WT}\NGF in brown.
(XLS)

Table S2 **Differentially modulated processes for each of the three different treatments, at 1 and 4 hours.** The items were selected from the Panther Database of Biological Processes (http://www.pantherdb.org/tools/genexAnalysis.jsp) using the Wilcoxon Rank-Sum Test (p-value<0.05) on two-columns tables including the list of differentially expressed genes and the related fold change values. The processes specific to each treatment and time point are highlighted in bold while the processes common to both the pro-NGF treatments (KR and wild type), separately at 1 h and 4 h, are highlighted in italic bold.
(PDF)
