# Supplementary figures and images for: NGF and proNGF Regulate Functionally Distinct mRNAs in PC12 Cells: An Early Gene Expression Profiling
Source: PLoS One. 2011 Jun 3;6(6):e20839. doi: 10.1371/journal.pone.0020839 (PMC3109000; doi:10.1371/journal.pone.0020839)

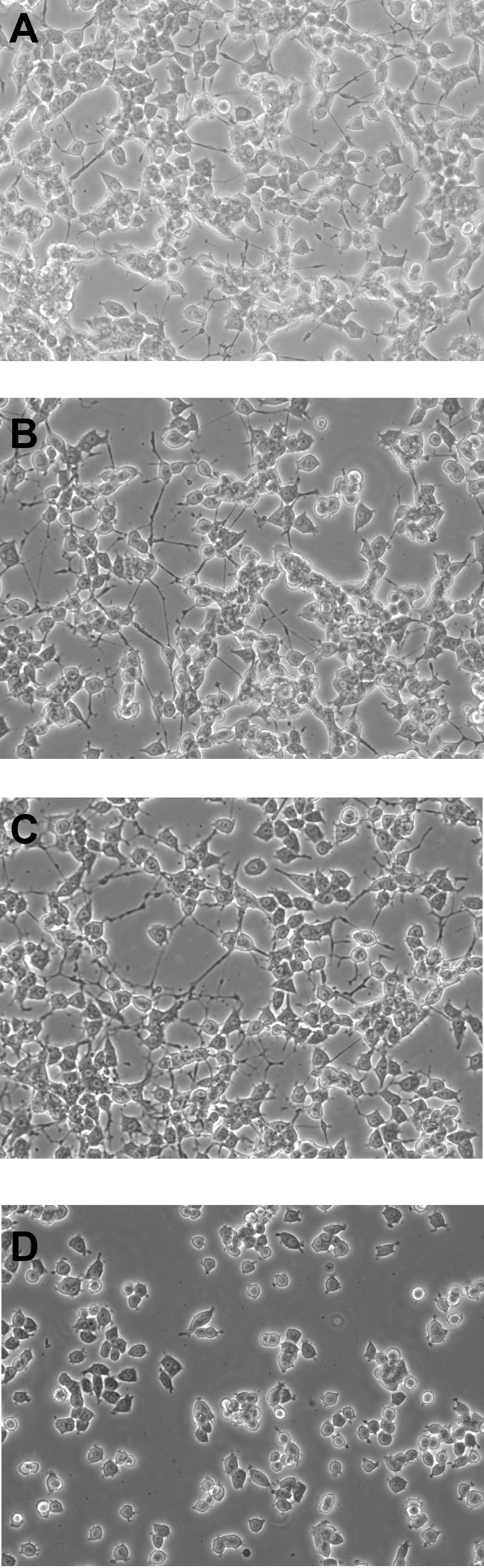

Supplement: Figure S1 — PC12 cells treated with the recombinant neurotrophins – 72 h after treatment. Panel A – 50 ng/mL of NGF. Panel B – 100 ng/mL of proNGF-WT. Panel C – 100 ng/mL of proNGF-KR. Panel D – Control without addition of neurotrophins. (TIF) [file pone.0020839.s001.tif]

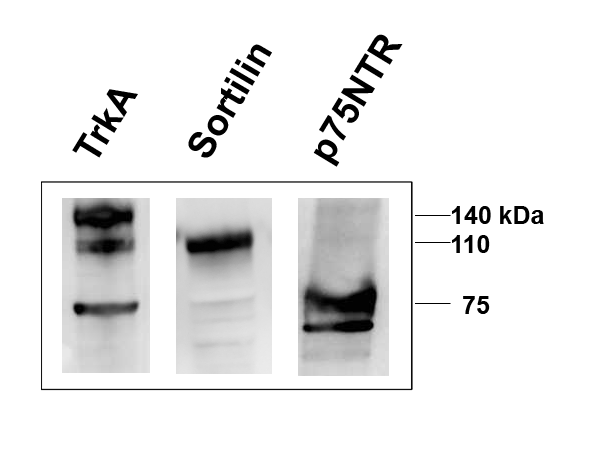

Supplement: Figure S2 — Receptors characterization in the PC12 SB subclone. PC12 cells lysate was subjected to Western blotting. The antibodies for the three receptors (TrkA, p75NTR and sortilin) were used. (TIF) [file pone.0020839.s002.tif]
