# Supplementary material for: NGF and proNGF Regulate Functionally Distinct mRNAs in PC12 Cells: An Early Gene Expression Profiling
Source: PLoS One. 2011 Jun 3;6(6):e20839. doi: 10.1371/journal.pone.0020839 (PMC3109000; doi:10.1371/journal.pone.0020839)
Supplement: Table S2 — Differentially modulated processes for each of the three different treatments, at 1 and 4 hours. The items were selected from the Panther Database of Biological Processes (http://www.pantherdb.org/tools/genexAnalysis.jsp) using the Wilcoxon Rank-Sum Test (p-value<0.05) on two-columns tables including the list of differentially expressed genes and the related fold change values. The processes specific to each treatment and time point are highlighted in bold while the processes common to both the pro-NGF treatments (KR and wild type), separately at 1 h and 4 h, are highlighted in italic bold. (PDF) [file pone.0020839.s004.pdf]

mNGF 1h

Biological Process	#	+/-	pvalue
Protein biosynthesis	71	+	7,65E-05
**mRNA transcription regulation**	**160**	**+**	**2,67E-04**
Synaptic transmission	37	-	4,09E-04
mRNA transcription	218	+	7,05E-04
Transport	154	-	9,12E-04
Nucleoside, nucleotide and nucleic acid metabolism	438	+	9,91E-04
Neurotransmitter release	18	-	1,68E-03
**Phospholipid metabolism**	**24**	**-**	**1,84E-03**
Ion transport	60	-	1,95E-03
Intracellular protein traffic	167	-	2,15E-03
Lipid, fatty acid and steroid metabolism	87	-	2,18E-03
**Cation transport**	**47**	**-**	**8,52E-03**
Lipid metabolism	14	-	8,54E-03
Cell structure and motility	120	-	1,12E-02
Neuronal activities	68	-	1,15E-02
**Embryogenesis**	**12**	**+**	**1,52E-02**
Cell motility	41	-	1,70E-02
**Inhibition of apoptosis**	**17**	**+**	**1,79E-02**
Neuromuscular synaptic transmission	6	-	2,01E-02
Pre-mRNA processing	56	+	2,10E-02
**Calcium ion homeostasis**	**8**	**-**	**2,59E-02**
**Nerve-nerve synaptic transmission**	**4**	**-**	**2,88E-02**
Receptor protein tyrosine kinase signaling pathway	30	-	2,99E-02
**Stress response**	**33**	**+**	**3,10E-02**
Regulated exocytosis	11	-	3,12E-02
Exocytosis	32	-	3,39E-02
Monosaccharide metabolism	9	-	3,51E-02
**Carbohydrate metabolism**	**62**	**-**	**3,64E-02**
**General vesicle transport**	**46**	**-**	**3,92E-02**
**MAPKKK cascade**	**29**	**+**	**4,05E-02**
Other pathways of electron transport	4	-	4,14E-02
Cell cycle	142	+	4,37E-02
**Protein glycosylation**	**29**	**-**	**4,48E-02**
Chromosome segregation	12	+	4,63E-02

proNGF 1h

proNGF wild type

Biological Process	#	+/-	pvalue
Protein biosynthesis	88	+	3,69E-07
Receptor protein tyrosine kinase signaling pathway	26	-	5,24E-04
Nucleoside, nucleotide and nucleic acid metabolism	465	+	7,04E-04
Synaptic transmission	34	-	8,03E-04
Chromosome segregation	20	+	2,43E-03
Cell cycle	163	+	2,49E-03
**Signal transduction**	344	-	3,54E-03
Neuromuscular synaptic transmission	6	-	3,61E-03
Exocytosis	29	-	6,15E-03
Regulated exocytosis	10	-	7,76E-03
Lipid, fatty acid and steroid metabolism	106	-	9,45E-03
Transport	161	-	9,75E-03
DNA replication	27	+	1,12E-02
**Cell surface receptor mediated signal transduction**	112	-	1,29E-02
Cell motility	40	-	1,33E-02
Pre-mRNA processing	65	+	1,39E-02
Cell structure and motility	130	-	1,48E-02
Other pathways of electron transport	4	-	1,69E-02
DNA metabolism	61	+	1,97E-02
General mRNA transcription activities	3	+	2,00E-02
**Protein modification**	202	-	2,35E-02
Neuronal activities	74	-	2,36E-02
Fatty acid metabolism	29	-	2,45E-02
**Developmental processes**	233	-	2,48E-02
Protein targeting and localization	31	+	3,44E-02
mRNA transcription	221	+	3,68E-02
Intracellular protein traffic	181	-	3,93E-02
**Other sensory perception**	3	-	4,03E-02
**Ligand-mediated signaling**	24	-	4,36E-02
Lipid metabolism	19	-	4,85E-02
**Protein folding**	34	+	4,89E-02

proNGF-KR

Biological Process	#	+/-	pvalue
Protein biosynthesis	64	+	7,26E-06
Lipid, fatty acid and steroid metabolism	87	-	7,42E-04
Synaptic transmission	37	-	2,86E-03
Cell structure and motility	121	-	3,57E-03
Transport	146	-	3,74E-03
Neuromuscular synaptic transmission	6	-	3,75E-03
Nucleoside, nucleotide and nucleic acid metabolism	406	+	4,95E-03
Other pathways of electron transport	4	-	5,60E-03
Neuronal activities	71	-	5,79E-03
Receptor protein tyrosine kinase signaling pathway	24	-	6,38E-03
Cell cycle	138	+	8,49E-03
Chromosome segregation	14	+	9,42E-03
Cell motility	39	-	1,24E-02
Intracellular protein traffic	159	-	1,45E-02
**Neurogenesis**	55	-	1,45E-02
Fatty acid metabolism	24	-	1,70E-02
DNA metabolism	52	+	1,94E-02
**Ectoderm development**	60	-	2,60E-02
Neurotransmitter release	17	-	2,86E-02
**Protein targeting**	22	+	2,95E-02
Protein targeting and localization	28	+	2,95E-02
Ion transport	61	-	3,18E-02
Monosaccharide metabolism	9	-	3,42E-02
Lipid metabolism	14	-	3,53E-02
DNA replication	23	+	4,19E-02
General mRNA transcription activities	3	+	4,97E-02

mNGF 4h

Biological Process	#	+/-	pvalue
**DNA metabolism**	**107**	**-**	**2,24E-07**
Cell structure and motility	211	+	2,15E-05
**DNA replication**	**49**	**-**	**3,95E-05**
**Cell motility**	**71**	**+**	**1,11E-04**
**Metabolism of cyclic nucleotides**	**13**	**-**	**1,11E-04**
**DNA repair**	**57**	**-**	**2,47E-04**
Chromatin packaging and remodeling	45	-	1,07E-03
**Pre-mRNA processing**	**68**	**+**	**1,59E-03**
**Cell structure**	**120**	**+**	**1,70E-03**
Chromosome segregation	23	+	5,62E-03
**Other coenzyme and prosthetic group metabolism**	**5**	**-**	**6,65E-03**
Synaptic transmission	51	-	6,93E-03
**Nerve-nerve synaptic transmission**	**8**	**-**	**1,14E-02**
**Homeostasis**	**46**	**-**	**1,47E-02**
Regulated exocytosis	16	-	1,73E-02
**Sex determination**	**4**	**+**	**1,95E-02**
**Intracellular signaling cascade**	**189**	**+**	**2,24E-02**
Protein biosynthesis	76	+	2,30E-02
**Carbohydrate metabolism**	**108**	**-**	**2,39E-02**
**B-cell- and antibody-mediated immunity**	**10**	**+**	**2,57E-02**
**Vision**	**17**	**-**	**2,70E-02**
**Oncogene**	**19**	**+**	**2,85E-02**
**mRNA splicing**	**51**	**+**	**3,27E-02**
**DNA recombination**	**19**	**-**	**3,43E-02**
**Regulation of phosphate metabolism**	**2**	**+**	**3,64E-02**
**Neuromuscular synaptic transmission**	**6**	**-**	**4,36E-02**
JNK cascade	14	+	4,67E-02
Cell proliferation and differentiation	164	+	4,74E-02
**Glucose homeostasis**	**9**	**+**	**4,85E-02**

proNGF 4h

proNGF wild type

Biological Process	#	+/-	pvalue
Protein biosynthesis	43	+	9,63E-04
Cell adhesion	35	-	1,74E-03
**Receptor protein tyrosine kinase signaling pathway**	23	-	6,85E-03
**Cholesterol metabolism**	10	+	8,13E-03
**Steroid metabolism**	12	+	9,69E-03
Cell structure and motility	95	-	1,01E-02
**Purine metabolism**	8	-	1,04E-02
Cell cycle	113	+	1,23E-02
General mRNA transcription activities	6	+	1,34E-02
Synaptic transmission	37	-	1,66E-02
**Signal transduction**	253	-	1,69E-02
Intracellular protein traffic	125	-	2,01E-02
**Ion transport**	42	-	2,12E-02
JNK cascade	10	+	3,03E-02
Cation transport	32	-	3,06E-02
Other nucleoside, nucleotide and nucleic acid metabolism	6	-	3,63E-02
Chromosome segregation	10	+	4,10E-02
Mitosis	36	+	4,24E-02
**Other protein metabolism**	3	+	4,48E-02
Stress response	33	+	4,59E-02
**Cell adhesion-mediated signaling**	15	-	4,79E-02
**General vesicle transport**	37	-	4,91E-02

proNGF-KR

Biological Process	#	+/-	pvalue
Cell cycle	124	+	1,08E-03
Cell adhesion	39	-	2,98E-03
Mitosis	38	+	4,30E-03
Chromosome segregation	11	+	1,04E-02
**Cell cycle control**	59	+	1,55E-02
Synaptic transmission	36	-	1,69E-02
**Chemosensory perception**	2	+	2,25E-02
JNK cascade	11	+	2,25E-02
**Olfaction**	2	+	2,25E-02
Protein biosynthesis	42	+	2,92E-02
**Neurotransmitter release**	15	-	2,97E-02
Intracellular protein traffic	115	-	2,98E-02
Regulated exocytosis	7	-	3,13E-02
Stress response	34	+	3,29E-02
Chromatin packaging and remodeling	28	-	3,39E-02
**Heart development**	3	+	4,42E-02
**Receptor protein serine/threonine kinase signaling pathway**	3	+	4,42E-02
General mRNA transcription activities	4	+	4,59E-02
Cell proliferation and differentiation	89	+	4,76E-02
Cation transport	37	-	4,86E-02
Other nucleoside, nucleotide and nucleic acid metabolism	6	-	4,86E-02
**Protein modification**	129	-	4,96E-02
